# Metabolic Capability and Phylogenetic Diversity of Mono Lake during a Bloom of the Eukaryotic Phototroph Picocystis sp. Strain ML

**DOI:** 10.1128/AEM.01171-18

**Published:** 2018-10-17

**Authors:** Blake W. Stamps, Heather S. Nunn, Victoria A. Petryshyn, Ronald S. Oremland, Laurence G. Miller, Michael R. Rosen, Kohen W. Bauer, Katharine J. Thompson, Elise M. Tookmanian, Anna R. Waldeck, Sean J. Loyd, Hope A. Johnson, Bradley S. Stevenson, William M. Berelson, Frank A. Corsetti, John R. Spear

**Affiliations:** a Department of Civil and Environmental Engineering, Colorado School of Mines, Golden, Colorado, USA; b Department of Microbiology and Plant Biology, University of Oklahoma, Norman, Oklahoma, USA; c Environmental Studies Program, University of Southern California, Los Angeles, California, USA; d Department of Earth Sciences, University of Southern California, Los Angeles, California, USA; e U.S. Geological Survey, Menlo Park, California, USA; f U.S. Geological Survey, Carson City, Nevada, USA; g Department of Earth, Ocean and Atmospheric Sciences, University of British Columbia, Vancouver, British Columbia, Canada; h Division of Chemistry and Chemical Engineering, California Institute of Technology, Pasadena, California, USA; i Department of Earth and Planetary Sciences, Harvard University, Cambridge, Massachusetts, USA; j Department of Geological Sciences, California State University Fullerton, Fullerton, California, USA; k Department of Biological Science, California State University Fullerton, Fullerton, California, USA; University of Tennessee and Oak Ridge National Laboratory

**Keywords:** Mono Lake, algal bloom, alkaline lake, geomicrobiology, metagenomics, transcriptomics

## Abstract

Algal blooms in lakes are often associated with anthropogenic eutrophication; however, they can occur without the human introduction of nutrients to a lake. A rare bloom of the alga Picocystis sp. strain ML occurred in the spring of 2016 at Mono Lake, a hyperalkaline lake in California, which was also at the apex of a multiyear-long drought. These conditions presented a unique sampling opportunity to investigate microbiological dynamics and potential metabolic function during an intense natural algal bloom. We conducted a comprehensive molecular analysis along a depth transect near the center of the lake from the surface to a depth of 25 m in June 2016. Across sampled depths, rRNA gene sequencing revealed that Picocystis-associated chloroplasts were found at 40 to 50% relative abundance, greater than values recorded previously. Despite high relative abundances of the photosynthetic oxygenic algal genus Picocystis, oxygen declined below detectable limits below a depth of 15 m, corresponding with an increase in microorganisms known to be anaerobic. In contrast to previously sampled years, both metagenomic and metatranscriptomic data suggested a depletion of anaerobic sulfate-reducing microorganisms throughout the lake's water column. Transcripts associated with photosystem I and II were expressed at both 2 m and 25 m, suggesting that limited oxygen production could occur at extremely low light levels at depth within the lake. Blooms of Picocystis appear to correspond with a loss of microbial activity such as sulfate reduction within Mono Lake, yet microorganisms may survive within the sediment to repopulate the lake water column as the bloom subsides.

**IMPORTANCE** Mono Lake, California, provides a habitat to a unique ecological community that is heavily stressed due to recent human water diversions and a period of extended drought. To date, no baseline information exists from Mono Lake to understand how the microbial community responds to human-influenced drought or algal bloom or what metabolisms are lost in the water column as a consequence of such environmental pressures. While previously identified anaerobic members of the microbial community disappear from the water column during drought and bloom, sediment samples suggest that these microorganisms survive at the lake bottom or in the subsurface. Thus, the sediments may represent a type of seed bank that could restore the microbial community as a bloom subsides. Our work sheds light on the potential photosynthetic activity of the halotolerant alga Picocystis sp. strain ML and how the function and activity of the remainder of the microbial community responds during a bloom at Mono Lake.

## Abstract (precis)

Mono Lake, California, provides a habitat to a unique ecological community that is heavily stressed due to recent human water diversions and a period of extended drought. To date, no baseline information exists from Mono Lake to understand how the microbial community responds to human-influenced drought or algal bloom or what metabolisms are lost in the water column as a consequence of such environmental pressures. While previously identified anaerobic members of the microbial community disappear from the water column during drought and bloom, sediment samples suggest that these microorganisms survive at the lake bottom or in the subsurface. Thus, the sediments may represent a type of seed bank that could restore the microbial community as a bloom subsides. Our work sheds light on the potential photosynthetic activity of the halotolerant alga Picocystis sp. strain ML and how the function and activity of the remainder of the microbial community responds during a bloom at Mono Lake.

## INTRODUCTION

**FIG 1:**
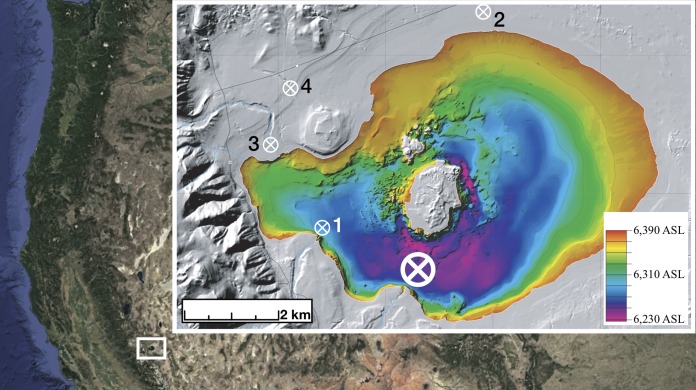
Overview of northeastern California, with Mono Lake inset. The approximate sampling location is shown by the large white circle/cross. Secondary sampling sites shown are sediment (1), well water (2), and Mill (3) and Wilson (4) creeks. Rush and Lee Vining creeks are not visible on the map. The overview image was captured from Google Earth/Landsat. The inset image was modified from U.S. Geological Survey Miscellaneous Field Studies map MF-2393 (56).

Mono Lake is a large hypersaline alkaline lake with a maximum depth of ≈50 m in the Mono Basin near the eastern foothills of the Sierra Nevada Mountains, California (Fig. 1). It formed from the remnant of Paleolake Russell (a Pleistocene glacial lake) and has existed as a closed basin for at least 50,000 years (1). Diversion of tributary streams to Mono Lake by the city of Los Angeles began in 1941 and resulted in a drop of over 13 m in lake level by 1978 (2), with a corresponding increase in water salinity from 48 g/liter to 81 g/liter by the 1990s (3) and a current alkalinity of 30,400 ppm HCO_3_^−^ (4). The steep decline in lake level also resulted in increasing concentrations of other solutes (including arsenic), resulting in unusual lake geochemistry and an absence of large macrofauna (e.g., fish) (5). Mono Lake is home to a photosynthetic eukaryotic alga, Picocystis (6), that is the primary food source of a brine shrimp endemic to the lake, Artemia monica (7). In turn, Artemia is a crucial food source for birds along the North American Pacific flyway (8, 9) where Mono Lake's microbial/eukaryotic ecosystem serves a unique, multicompartment, interlinked ecosystem role.

Picocystis is a genus of phototrophic alga, previously characterized in other saline or alkaline environments (10, 11). Picocystis sp. strain ML, identified in Mono Lake (6), is a near relative of Picocystis salinarium, isolated from the San Francisco Salt Works in a high-salinity (∼85‰) pond (10). In addition to P. salinarium, other near relatives have been identified from hypersaline environments in inner Mongolia (12). Picocystis sp. strain ML at Mono Lake is responsible for 100 mmol of C m^−2^ day^−1^ of the primary productivity in the lake (6). The population density of Picocystis varies throughout the year, often reaching a maximum in early spring before falling as Artemia shrimp graze on the algal cells, reproduce, and greatly reduce their number as measured by cell count (5). Although Picocystis is nitrogen limited, if sufficient concentrations of ammonia are present during lake mixing and turnover (5), a bloom can occur often coinciding with periods of lake anoxia (6). Elevated concentrations of chlorophyll *a* and Picocystis are commonly detected below the oxycline (6), but it is unknown if Picocystis is actively producing photosynthetic pigments and photosynthesizing under low-light conditions *in situ* at depth. If Picocystis is capable of phototrophic growth below the oxycline, particularly during a bloom when it is found in high densities at all depths, localized production of oxygen may disrupt anaerobic microbial communities in the bottom waters of Mono Lake.

How the bacterial and archaeal communities of Mono Lake respond during periods of algal bloom remains an open question. Prior to 2017, microbial community surveys of the lake during meromixis were carried out using only 16S rRNA gene clone library sequencing or denaturing gradient gel electrophoresis (DGGE) (13) that provide limited coverage (14). More recent work using 454 pyrosequencing (15) at Mono Lake provided additional diversity information for the lake during the onset of meromixis; however, the PCR primers chosen for previous high-throughput and clone library-based amplicon surveys at Mono Lake were potentially biased against certain bacterial phyla and lacked the ability to concurrently amplify the eukaryotes (16, 17). With more recent primers for Illumina-based amplicon sequencing (17), a more accurate representation of the lake microbial community is now possible. Furthermore, community distribution and profiling within Mono Lake during monomixis, or mixing of lake waters during a single time in a year, has yet to occur. Metagenomic and transcriptomic sequencing can also provide additional insight into the genetic activity of Mono Lake. Recently, genes associated with sulfate reduction were identified below the oxycline (≈15 m) while the lake was meromictic (i.e., stratified) (18). In that study (18), Edwardson and Hollibaugh conducted transcriptional profiling with the same samples sequenced for rRNA gene analyses in another recent study (15), which describes the microbial activity from the surface to below the oxycline. However, these analyses were heavily focused on the bacterial and archaeal components of Mono Lake, ignoring the eukarya.

A molecular description of the eukaryote responsible for much of the primary productivity in Mono Lake remains lacking from recent research. A description of how the green alga responsible for this primary productivity, Picocystis sp. strain ML (phylum Chlorophyta), is distributed within Mono Lake during a bloom and its impact on the ecophysiology of the lake are of crucial importance to ensure that a critical food source for migratory macrofauna is not lost. Furthermore, there is an outstanding question of whether or not sulfate-reducing microorganisms previously identified alongside strictly anaerobic Clostridia at a depth of 15 m below the oxycline (18) remain during a bloom of phototrophic alga that produces oxygen throughout the water column.

Mono Lake entered into a period of monomixis in 2012 corresponding to the onset of a near-record drought in the eastern Sierra. This resulted in a subsequent bloom of Picocystis in 2013 that failed to subside over the next 3 years (19) and corresponded with a near-record low of Artemia present within the lake in 2015. Lake clarity was at near-record lows, and measured chlorophyll *a* concentrations were high in 2016 (19). Here, we describe the effects of an algal bloom during a period of intense drought within the Mono Lake watershed during the summer of 2016 on the distribution and abundance of the bacterial, archaeal, and eukaryotic planktonic microbial communities and compare the results to those of previously sampled years within the lake (15, 18). This analysis was conducted via a combination of 16S/18S rRNA gene sequencing, metagenomics, and metatranscriptomic sequencing coupled to measurements of water chemistry and other physical measurements within the lake. The possibility that the microbial community within the lake could be repopulated by the sediment, groundwater, and the influent streams that feed Mono Lake is also addressed by 16S/18S rRNA gene sequencing. Finally, we sought to determine if Picocystis sp. strain ML is transcriptionally active under extremely low light levels in the lake.

## RESULTS

### Major ion chemistry and microbial rRNA gene copy number within Mono Lake.

**FIG 2:**
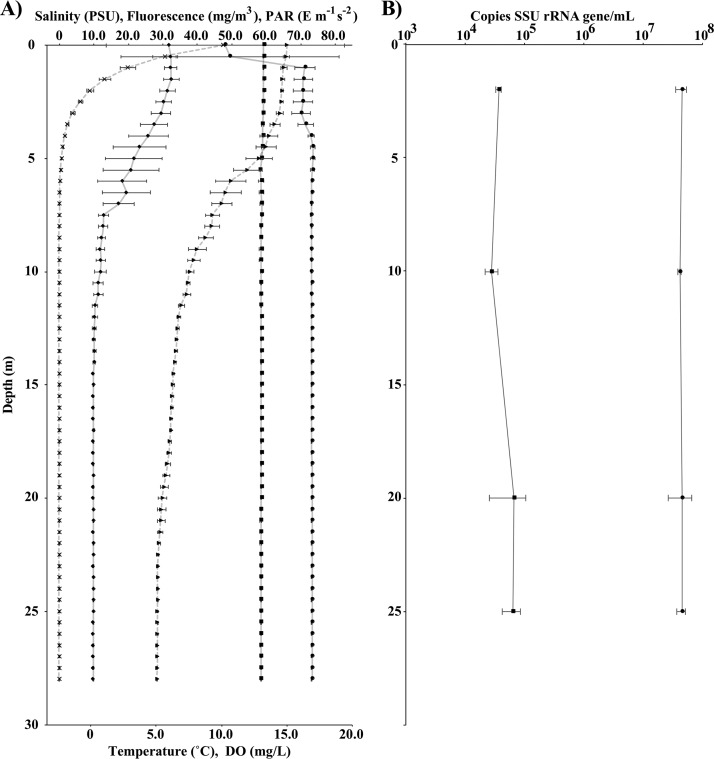
(A) CTD measurements taken during 2016 sampling with salinity (squares), fluorescence (circles), and PAR (crosses) shown on the upper axis; temperature (triangles) and dissolved oxygen (diamonds) are shown on the lower axis. Points are half-meter averages, with standard deviations shown. For clarity, lines connecting temperature and PAR are dashed. PSU, practical salinity units. (B) Quantification of 16S (filled circles) and 18S (filled squares) rRNA gene copy numbers at discrete sampling depths of 2, 10, 20, and 25 m. Error bars represent the mean standard deviation of triplicate biological and triplicate technical replicates.

Water was sampled along a continuous transect for temperature, photosynthetically active radiation (PAR), dissolved oxygen (DO), and fluorescence (Fig. 1). At depths between 5 and 15 m, the water temperature decreased from ≈15 to ≈7°C. DO and PAR declined rapidly within the first 10 m, yet fluorescence was above detectable limits throughout the sampled depths (Fig. 2A). Discrete samples were also taken for microbial density and water chemistry at the surface and at depths of 2, 10, 20, and 25 m. Microbial density estimated by bacterial and archaeal 16S rRNA gene copy number varied by less than 10% from 2 to 25 m. In contrast, a eukaryotic 18S rRNA gene copy number maximum was present at 20 and 25 m (Fig. 2B). Major anions, including sodium (Na^+^), were consistent, and levels were near previously reported values (Table 1). Only minimal differences in anion or cation concentrations were detected within Mono Lake. Nitrate, nitrite, and sulfate were elevated at 10 m relative to levels at 2, 20, and 25 m. No phosphate was detectable by ion chromatography (IC) from 2 to 25 m within Mono Lake though surface water taken near shore had an average value of 0.02 mM (Table 1). Average total dissolved phosphorus (potentially including phosphate and organophosphorus) measured by inductively coupled plasma atomic emission spectroscopy (ICP-AES) ranged from 0.59 to 0.63 mM from the surface to a depth of 25m, respectively (Table 1). Most major anions and cations and dissolved inorganic carbon were below detectable limits in the sampled influent stream water and nearby well water, with the exception of calcium, which was elevated relative to levels in Mono Lake water samples (Table 1). Individual replicate results for ICP-AES and IC are shown in Table S1 in the supplemental material.

**TABLE 1:** Measured geochemical parameters from the water column, as well as nearby streams and well water, representing subsurface water below Mono Lake

Analyte	Value by sample depth (mM)^*a*^					Value by sample source (mM)^*a*^				
	Surface	2 m	10 m	20 m	25 m	Well	Lee Vining	Mill	Rush	Wilson
As^*b*^	0.19 ± 0.01	0.18 ± 0.00	0.19 ± 0.01	0.17 ± 0.00	0.20 ± 0.03	BDL	BDL	BDL	BDL	BDL
Br^*b*^	0.76 ± 0.09	0.98 ± 0.00	1.10 ± 0.05	1.97^*e*^	0.96 ± 0.01	BDL	BDL	BDL	BDL	BDL
Ca^*b*^	BDL	BDL	BDL	BDL	BDL	3.2 ± 3.4	2.7^*e*^	1.1 ± 1.7	4.5 ± 4.0	1.6 ± 2.4
Cl^−^^*c*^	57 ± 4.1	588 ± 1.9	695 ± 35	585 ± 7.6	577 ± 7.3	0.24 ± 0.01	0.02 ± 0.00	0.01 ± 0.00	0.07 ± 0.00	0.01 ± 0.00
F^−^^*c*^	2.7 ± 0.34	3.6 ± 0.02	4.1 ± 0.17	3.5 ± 0.03	3.5 ± 0.06	0.02 ± 0.00	BDL	0.01^*e*^	BDL	BDL
Fe^*b*^	0.01	0.01	BDL	BDL	BDL	0.02	BDL	BDL	BDL	0.01
K^*b*^	37 ± 7.1	39 ± 2.0	38 ± 4.0	37 ± 5.5	41 ± 3.8	0.24 ± 0.21	BDL	BDL	BDL	BDL
Mg^*b*^	1.9 ± 1.4	1.7 ± 1.2	1.0 ± 0.02	1.0 ± 0.10	1.4 ± 0.30	2.8 ± 4.2	1.5^*e*^	0.14^*e*^	0.34^*e*^	5.1^*e*^
Na^*b*^	1,030 ± 10	874 ± 13	866 ± 74	696 ± 286	892 ± 59	4.2 ± 0.31	0.29 ± 0.26	0.33^*e*^	0.37 ± 0.17	0.55 ± 0.60
NO_2_^*c*^	0.37^*e*^	BDL	0.77^*e*^	BDL	BDL	BDL	BDL	BDL	BDL	BDL
NO_3_^*c*^	0.01^*e*^	0.03^*e*^	0.15^*e*^	0.03^*e*^	0.09^*e*^	0.01 ± 0.00	0.01 ± 0.00	BDL	BDL	BDL
P^*b*^	0.59 ± 0.08	0.62 ± 0.05	0.61 ± 0.03	0.59 ± 0.04	0.63 ± 0.07	BDL	BDL	BDL	BDL	BDL
PO_4_^*c*^	0.02^*e*^	BDL	BDL	BDL	BDL	BDL	BDL	BDL	BDL	BDL
S^*b*^	111 ± 13	125 ± 1.2	124.68 ± 2.21	120.48 ± 3.6	130 ± 4.7	0.29 ± 0.11	0.05 ± 0.04	0.17 ± 0.06	0.16 ± 0.12	0.25 ± 0.10
SO_4_^*c*^	122 ± 1.1	113 ± 0.71	136.73 ± 9.30	113.62 ± 1.7	112 ± 1.1	0.25 ± 0.01	0.05 ± 0.00	0.13 ± 0.00	0.05 ± 0.00	0.15 ± 0.00
DIC^*d*^	NA	313	300	322	318	5.19	NA	NA	NA	NA

a Values are the average of triplicate samples, unless otherwise noted. BDL, below detectable limit; NA, not available (sample not measured).

b Measured using ICP-AES.

c Measured using ion chromatography.

d Dissolved inorganic carbon (DIC) reported from a single sample per site.

e Measurement from one or two samples. No standard deviation was calculated.

### Bacterial and eukaryotic microbial communities of Mono Lake, sediment, and influent streams.

**FIG 3:**
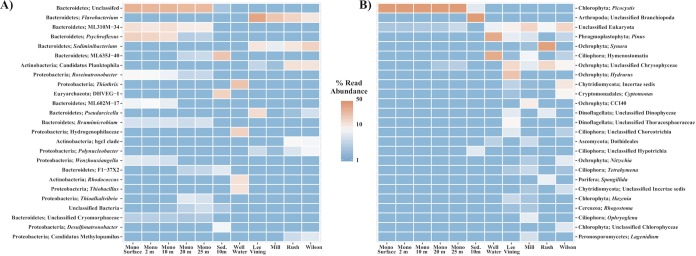
Heat map of the top 25 OTUs within the bacteria/archaea (A) and eukaryotes (B). OTUs are named by phyla and the most likely genera.

**FIG 4:**
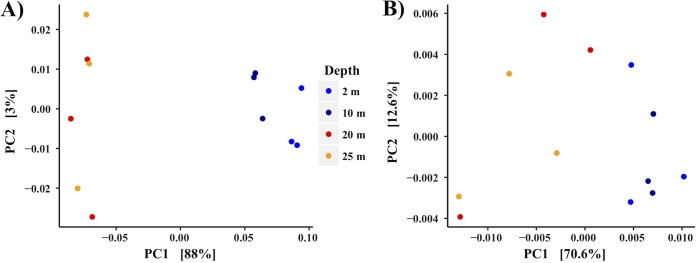
Principal coordinate analysis (PCoA) ordination of bacterial/archaeal (A) and eukaryotic (B) communities of water samples taken at Mono Lake. Ordination is based on a weighted UniFrac distance matrix.

Water and sediment samples, as well as water samples from influent streams and groundwater, were PCR amplified to determine their bacterial, archaeal, and eukaryotic community composition. After quality control, a total of 694,948 16S rRNA gene sequence reads were obtained for an average of 13,364 reads per sample, clustering into 831 operational taxonomic units (OTUs); summary statistics are found in Table S2. Chloroplast sequences were abundant across all lake water samples (Fig. S1) and were removed from analysis to more clearly visualize the remaining bacterial and archaeal microbial communities (Fig. 3A). Both the bacterial and archaeal communities differed in structure above and below the oxycline at 10 m (Fig. 3A). Samples taken from sediment were distinct in bacterial, archaeal, and eukaryotic community structures from those in the sampled water column. Two OTUs most closely related to genera within the order Bacteroidetes decreased in relative abundance steadily with depth (Psychroflexus and ML602M-17), whereas unclassified Bacteroidetes remained relatively constant in abundance throughout the water column (Fig. 3A). An OTU most closely related to the genus Thioalkalivibrio increased in abundance as depth increased. Unique to the sediment were Euryarchaeota (Thermoplasmata, DHVEG-1) and the bacterial genus Desulfonatronobacter. An increase in the relative abundance of chloroplast sequence was noted at 20 m, increasing from ≈40% at the surface to ≈48% at 10 m and then to ≈62% relative abundance at 20 m (Fig. S1). Nearby well water taken to compare to lake water samples contained an abundant population of OTUs most closely related to sulfur-oxidizing Proteobacteria including Thiothrix and Thiobacillus, as well as Actinobacteria (Rhodococcus), and an abundant unclassified OTU within the Hydrogenophilaceae. Mono Lake influent stream water samples collected and examined from Rush, Mill, Lee Vining, and Wilson creeks were distinct from samples taken from the lake itself, with the Flavobacteria, Sediminibacterium, and the hgcI clade of the Actinobacteria being the most abundant OTUs across all stream samples (Fig. 3A). Mill samples were outliers to other stream water samples, lacking abundant populations of Actinobacteria (“Candidatus Planktophila” and hgcI clade) and having a lower abundance of the Sediminibacterium than samples from Lee Vining, Rush, and Wilson streams. Community membership and distribution in the lake water column profile samples were significantly influenced (*P* = 0.002, *R*^2^ = 0.90) by depth, and the transition to anoxia was visualized by weighted UniFrac principal coordinate analysis (PCoA) ordination and a corresponding Adonis test (Fig. 4A).

The eukaryotic community contained far fewer OTUs than the observed bacterial and archaeal communities. Within the water column at Mono Lake, an almost homogenous distribution of OTUs most closely related to the genus Picocystis was observed at all depths, with a maximum of 97.9% relative abundance at a depth of 10 m (Fig. 3B) during the sampled bloom event of 2016. Within the sediment, an OTU of unclassified Branchiopoda was most abundant, comprising 90.9% of all sediment eukaryotic sequence. BLAST results of this OTU suggest that it is most likely Artemia monica, endemic to Mono Lake, although because of the short sequence read length of 250 bp the identification is ambiguous. Influent stream water samples were distinct from samples of the water and sediment of Mono Lake, with few overlapping OTUs among the samples (Fig. 3B). Specifically, multiple OTUs most closely related to the Ochrophyta (heterokont alga), Ciliophora, and Chytridiomycota were unevenly distributed across the stream and well water sampled. Community membership and distribution within the water column in Mono Lake were significantly influenced (*P* = 0.017, *R*^2^ = 0.61) by depth although less significantly than for the bacteria and archaeal communities (*P* = 0.002, *R*^2^ = 0.90) (Fig. 4B).

### Metagenomic and transcriptomic profiling of Mono Lake and sediments.

The abundance of sulfate (>100 mM) and the lack of oxygen below the 10-m oxycline beg the question as to whether active sulfate reduction is occurring in the dissolved organic carbon (DOC)-rich waters of Mono Lake. No sulfite oxidase (*sox*) genes were identified; however, genes for the complete reduction of sulfate to sulfide were identified in the sediment metagenome, and genes for reverse-dissimilatory (or oxidative) sulfite reductases (*dsrA*) were identified in water column metagenomes. Only reverse, or oxidative, *dsrA* genes were identified in the water column metagenomes. Dissimilatory sulfite reductase genes within the sediment metagenome had high (>80%) homology to known deltaproteobacterial sulfate-reducing microorganisms. Reductive *dsrA* and *dsrB* genes were identified within the water column, putatively via BLAST, and were most closely related to known *Thioalkalivibrio dsrA* and *dsrB* genes. Sulfite reductase genes did not appear to be expressed within the metatranscriptome (Table S4). Nitrate and nitrite reductases were found at 20 and 25 m and within the sediment, while nitric oxide reductase (*nor*) was identified only within the sediment (Table S3). Genes associated with nitrogen fixation, including *nifH*, *nifD*, and *nifK*, were found at 20 m within the water column and within the sediment metagenome. No genes associated with ammonium oxidation by bacteria or archaea (AOB/AOA) were identified. Formate-dependent nitrite reductases were identified as both genes and transcripts. A comprehensive list of identified transcripts is available in Table S4 in the supplemental material. A summary of assembly statistics for sediment and water samples is available in Table S3.

### MAGs of Mono Lake and sediment.

**FIG 5:**
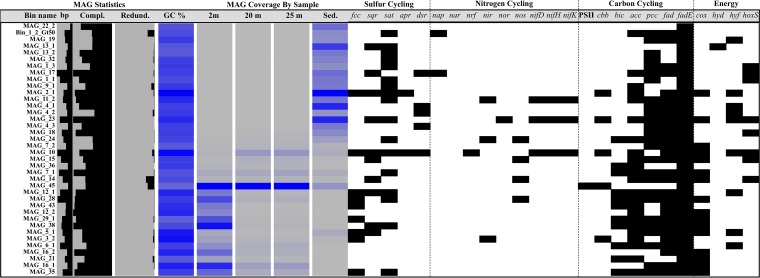
Overview of detected MAGs across sampled metagenomes. An increase in the color intensity from gray to blue corresponds to an increase in the coverage of each MAG within each sample. Estimates of GC content, completeness (Compl.), and contamination (or redundancy [Redund.]) of each MAG are also given. Presence (black) of key genes related to sulfur, nitrogen, and carbon cycling, as well as respiration is also shown. *fcc*, sulfide dehydrogenase; *sqr*, sulfide-quinone reductase; *sat*, sulfate adenylyltransferase; *apr*, adenosine-5-phosphosulfate reductase; *dsr*, dissimilatory sulfite reductase; *nap*, periplasmic nitrate reductase; *nar*, nitrate reductase; *nrf*, nitrite reductase; *nir*, nitrite reductase; *nor*, nitric oxide reductase; *nos*, nitric oxide synthase; *nifD*, nitrogenase molybdenum-iron protein alpha chain; *nifH*, nitrogenase iron protein 1; *nifK*, nitrogenase molybdenum-iron protein beta chain; PSII, photosystem II genes *psbA* and *psbB*; *cbb*, ribulose 1,5-bisphosphate carboxylase/oxygenase; *bic*, bicarbonate transporter; *acc*, acetyl-coenzyme A carboxylase; *pcc*, propionyl-coenzyme A carboxylase; *fad*, long-chain fatty acid-coenzyme A ligase; *fadE*, acyl-coenzyme A dehydrogenase; *cox*, cytochrome *c* oxidase; *hyd*, hydrogenase I; *hyf*, hydrogenase-4; *hoxS*, bidirectional NiFe hydrogenase.

After refinement binning, a metagenomic analysis identified 80 metagenome-assembled genomes (MAGs) with various levels of completion and contamination (Table S3). Of the 80 identified MAGs, 38 were greater than 50% complete and contained less than 10% contaminating DNA sequence. A total of 21 MAGs contained rRNA gene sequence, and a putative identification was produced from these data (Fig. S2). As with the rRNA gene sequencing data, MAGs indicated that microbial community composition shifted by depth and correlated with the decline in oxygen at 10 m (Fig. 5). A large number of MAGs were unique to the sediment, including a euryarchaeon most closely related to the Thermoplasmatales (Fig. 5; see also Fig. S2 in the supplemental material). No archaea were found in abundance throughout the sampled water column, and no genes associated with the production of methane (archaeal methanogenesis) were identified. Multiple MAGs were recovered from uncultivated orders within the Actinobacteria, Gammaproteobacteria, and Bacteroidetes (Table S3), including MAGs with 16S rRNA gene sequences previously identified by rRNA gene clone library sequencing at Mono Lake, such as ML602J-51 (13). A summary of each genome is available in Table S3 and Fig. 5. No MAGs were identified with the genes required for sulfate reduction, with only reverse *dsr* genes for sulfur oxidation found in MAGs. Nitrogen fixation genes (*nifH*, *nifD*, and *nifK*) were identified within three MAGs, two within the Gammaproteobacteria (bins 10 and 23) and a single unclassified bin (bin_11_2). One bin (bin 45) contained photosystem II-associated genes, identified within the Epsilonproteobacteria (Fig. 5 and Table S3). Three MAGs were identified in a separate filtered metagenomic data set. A 1,469-bp 16S rRNA gene sequence was identified by BLAST within the largest eukaryotic MAG as being closely related to that of known Picocystis sp. chloroplasts. Additionally, two 28S rRNA gene fragments were also identified. The putatively identified Picocystis MAG was estimated to be 49.40% complete, with a total length of 11.14 Mbp, and 2.41% redundant. Putative identification of sequence by BLASTx within the MAG returned homology to other known algae. The annotated genome contained no genes related to sulfur cycling or other incomplete metabolic cycles (Fig. S3) although completion estimates suggest that only half of its genome was identified. Summary assembly statistics, including completion and redundancy estimates of each putative eukaryotic MAG, are available in Table S3.

### Photosynthesis transcripts are detectable at 25 m.

Assembly of transcriptomes from 2 m and 25 m resulted in 113,202 coding sequences and 111,709 annotated protein coding genes (Table S4). No transcripts were identified with homology to known dissimilatory sulfite reductases in contrast to results for previously sampled years (11). A total of 3,117 genes were differentially expressed (*P* < 0.05, false discovery rate [FDR] corrected) between 2 m and 25 m (Table S4). Genes associated with photosystem I and II pathways were expressed at both sampled depths (Table 2; see also Table S4). Normalized expression values (reported as the number of transcripts per kilobase million, or TPM) for photosystem I and II, including those of *psaA* and *psaB*, *psbA* and *psbB*, and *psbC* transcripts, were at higher levels at a depth of 25 m than at 2 m (Table 2). Transcripts for multiple housekeeping genes, including *gyrA* and *gyrB*, the glyceraldehyde-3-phosphate dehydrogenase (GAPDH) gene, and the tubulin subunit alpha gene were either at similar numbers for the different depths or lower at 25 m, indicating similar or lower numbers of cells at depth, despite higher transcript levels (Table 2; see also Table S4). In addition, several light-independent protochlorophyllide reductase transcripts were differentially expressed at 25 m, while no transcripts related to chlorophyll production were differentially expressed at 2 m (Table S4).

**TABLE 2:** Mean expression values of select genes identified associated with photosynthesis

Gene^*a*^	Avg expression (TPM) at:		Log_2_ fold change	*P* value (FDR)
	25 m	2 m		
*psaA*	155.4	80.4	1.0	0.008
*psaA*	209.7	86.8	1.3	0.0001
*psaB*	247.0	123.0	1.0	0.002
*psbA*	563.7	176.7	1.7	<0.0001
*psbB*	241.1	97.4	1.3	<0.0001
*psbB*	165.4	74.8	1.1	0.001
*psbC*	183.5	84.1	1.1	<0.0001
*psbY*	23.5	18.4	0.3	>0.05
TUBA gene	1.7	4.5	−1.5	>0.05
*gyrA*^*b*^	4.2	4.4	−0.03	
*gyrB*^*b*^	5.2	5.5	−0.04	
GAPDH gene^*b*^	5.2	9.4	−0.86	

a TUBA, tubulin alpha-1 chain; GAPDH, glyceraldehyde 3-phosphate dehydrogenase.

b Shown as an average expression value of all annotated transcripts at each sampled depth.

## DISCUSSION

Beginning in late 2012, Mono Lake exhibited signs of persistent Picocystis blooms that corresponded to developing drought conditions within the region. Subsequently, from 2013 to 2016, both lake clarity and Artemia abundance declined dramatically (19). Surface concentrations of chlorophyll *a* had been relatively stable over the past 2 decades, with an average 3.8 μM in July (1994 to 2013), yet in the 2016 algal bloom, concentrations were 10 times higher, 33.9 μM (19). The elevated chlorophyll *a* concentration and Secchi disk values (indicative of lake clarity) above 1 m are consistent with a bloom of Picocystis in Mono Lake. In addition, the relative abundances of microorganisms presented here and the well-mixed major ions of Mono Lake relative to levels reported in previous work (13, 18) indicated that Mono Lake was well within a bloom of the green alga Picocystis and a concurrent period of monomixis. Genes required for sulfate reduction to sulfide were detected only in the sequenced lake sediment, while both metagenomic and 16S rRNA gene sequencing indicated a nearly complete loss of the detectable anaerobic sulfate-reducing potential within the water column of Mono Lake. Instead, a mixed algal and facultatively anaerobic microbial community was present below the detectable oxycline at 10 m, more similar to the near-surface microbial community than previously reported (18). It is yet unknown how the microbial community of Mono Lake will rebound after such a significant algal bloom and a decline in the population of Artemia within the lake.

Our survey allowed a comprehensive evaluation of the genomic potential and expressed genes associated with metabolic processes throughout the water column. Dissimilatory nitrate reduction to ammonium (DNRA) appeared active, with formate-dependent cytochrome *c* nitrite reductases detected within the transcriptome (see Table S4 in the supplemental material) and formate-dependent nitrite reductase subunits detected within the assembled metagenomes (Table S3). No genes associated with bacterial ammonium oxidation (AOB) were identified, in contrast to findings in previous years (20), in either the transcriptome or metagenome, suggesting that the ammonia produced within the lake was assimilated, likely by the dense population of growing Picocystis. In addition to nitrate reduction, another key anaerobic respiratory process, sulfate reduction, was largely absent from the water column.

Previous work during meromixis/nonbloom intervals has shown that sulfate reduction is a key respiratory process in the Mono Lake water column, supporting the growth of multiple species of sulfide-oxidizing aerobic microorganisms above the oxycline (18). We found that microorganisms capable of sulfate reduction were identified only in sediment metagenomic samples during the 2016 bloom. Dissimilatory-type reverse sulfite reductases associated with sulfur oxidizing gammaproteobacterial (21) taxa were identified at 20 and 25 m, but no reductive sulfite reductases were found in sequenced water samples. Taxa known to reduce sulfate were also identified only by 16S rRNA gene sequencing within the water column in stark contrast to previously sampled years (18, 22). Instead, the most abundant microorganisms with identifiable *dsrA* and *dsrB* gene clusters were reverse-*dsr* type reductases identified previously in the gammaproteobacterium genus Thioalkalivibrio (21). While lake sulfate reduction rates are typically very low (23), our data suggest that microorganisms known to reduce sulfate, as well as key genes in dissimilatory sulfate reduction, are lost in the water column during a bloom. It is also possible that rather than a total loss of this metabolic capability, there is instead a heavy reduction in the number of microorganisms that falls below the level of detection of our sequencing effort. It is likely that during a bloom, sulfate reduction is repressed as more oxidizing conditions are present throughout the water column due to an increased abundance of oxygenic photosynthetic algae. Members of the Bacteroidetes were in high abundance throughout the water column, including OTUs most closely related to ML310M-34, a Bacteroidetes identified previously in Mono Lake (13), which remained abundant through the water column while Psychroflexus decreased in abundance from 2 to 25 m as oxygen levels declined. The eukaryotic microbial community was more evenly distributed throughout the water column, with Picocystis detected in nearly equivalent relative abundances throughout the column (Fig. 3B), agreeing with reported chlorophyll levels (19) as well as fluorescence values measured as a part of this study (Fig. 2A).

Eukaryotic 18S rRNA gene copy number was greater at 20 and 25 m than above the oxycline by approximately 40%. The results were similar to previous estimates of Picocystis biomass during bloom events in which the greatest concentrations of Picocystis were near the bottom of the lake (6). Artemia grazing pressure was unusually low during 2016, which then allowed for the increase in Picocystis abundance throughout the sampled water column and accounted for the similarly low visibility (Secchi disk) readings. Additional primary productivity in the lake could also account for the oxycline shallowing from a depth of 15 m in 2013 (13, 18) to a 10-m depth in July 2016. This expansion of anoxic waters likely limits Artemia populations from grazing on Picocystis, resulting in a lower total amount of biomass of Artemia available for migratory birds along the North American Pacific flyway. Lake temperature decreases at the surface relative to temperatures reported in previous studies may also slow the metabolism of Artemia, resulting in reduced fecundity and increased mortality (24, 25). A decline in the Artemia population could also impact bird mortality though this was outside the scope of this study and should be investigated at a later date.

Evidence exists that Picocystis sp. strain ML appears capable of photosynthesis under very low light conditions near the bottom of Mono Lake. Previous work suggested that Picocystis sp. strain ML is capable of growth with less than 0.1% of incident summertime solar irradiance, which corresponded with increased concentrations of chlorophyll below 15 m at Mono Lake (6). In our work, chloroplast 16S rRNA gene sequence was most abundant at 20 m, corresponding to a peak in total 16S rRNA copy number (Fig. 2B). 18S rRNA gene sequences identified as Picocystis sequences were most abundant at 10 m, yet chloroplast relative abundance peaked at 20 m, near previously recorded peak depths in other recorded bloom events (6). Despite the high relative abundance of Picocystis throughout the water column, the isolation and characterization of the Picocystis genome remain elusive. Metagenomic binning resulted in a partial MAG of ≈50% completion. Genome sequencing of Picocystis, recently isolated and sequenced twice independently (Ronald Oremland, personal communication), will allow for its genome to be removed from subsequent sequencing efforts, which will simplify assembly and enhance the resolution of bacterial and archaeal binning efforts in the future, yielding a better understanding of the microbial community responsible for the diverse metabolic potential in both the sediments and water of Mono Lake.

Our *de novo* transcriptomic assembly was able to recover Picocystis chloroplast-associated transcripts in addition to other microbial transcripts. At a 25-m depth, differential expression of photosystem II was observed (Table 1; see also Table S4 in the supplemental material), suggesting active photosynthesis under extremely low light conditions. Recently, photosynthesis in a microbial mat was shown to be capable under extremely low light conditions although in a bacterial system (26). It is possible that Picocystis is sinking from 2 m, settling near 25 m, and artificially increasing the estimates of eukarya and associated transcription estimates at depth. Housekeeping genes including GAPDH, known to work well in the normalization of algal transcriptome data (27), and also including *gyrA* and *gyrB* were expressed below a 1-log_2_-fold change. Another eukaryote-associated housekeeping gene, TUBA (27), was expressed above a 1.5-log_2_-fold change but was not significantly differentially expressed. All housekeeping gene expression levels were far below those of the identified photosynthesis transcripts (Table 2), and genes identified as eukaryotic in origin were not differentially expressed (Table S4), suggesting that the numbers of transcriptionally active eukarya throughout the water column are similar. The differential transcription of chloroplast-related chlorophyll proteins is in line with previous research that showed a 10-fold increase in the amount of photopigment present at low light (6) and suggests that under extreme-low-light conditions, photosynthesis may still occur. Still, the difference could be accounted for by falling biomass in such an extreme bloom rather than by an active photosynthetic metabolism as alga could remain metabolically active having just landed in the sediment zone. Further sampling during a bloom event, including rate measurements of oxygen production, photosynthesis, and pigments, may help to resolve this question. Additional laboratory examination of Picocystis in isolation under extremely low light conditions must also be carried out before this finding can be confirmed.

During algal bloom, Mono Lake experiences significant shifts in both the bacterial and archaeal microbial communities and in its metabolic potential from nonbloom years (15, 18). Picocystis was present throughout the water column with photosystem I and II transcripts identified, even at a 25-m depth. While Picocystis bloomed throughout Mono Lake, there was also a loss of detectable sulfate-reducing microorganisms. The lack of detectable sulfate reduction at and below 20 m within Mono Lake is in contrast to findings of previous work and is possibly linked to the intense drought experienced by Mono Lake from 2012 to 2016. During a multiyear drought, anaerobic microorganisms may survive within the underlying sediment even after they are killed off within the water column. By sequencing nearby sediment, we have shown that even if sulfate reduction is temporarily lost in the planktonic community of Mono Lake, the sediment may act as a seed bank or refugia for organisms capable of this and likely for other necessary metabolisms dependent upon overlying water/lake conditions (28). Alternatively, the sulfate-reducing microorganisms may find a better reduced substrate or fewer inhibitors in the sedimentary environment. Furthermore, the recovery of microbial populations within Mono Lake must come from its sediment or underlying groundwater, not from the streams that feed it as no overlapping taxa exist from the influent streams (Fig. 2). While humans have not added significant amounts of nutrients to Mono Lake, they have withdrawn large quantities of water, possibly exacerbating drought conditions over the years (2). Establishing if and how the chemistry and microbiota of Mono Lake recover after monomixis, drought, and algal bloom should be the focus of future work. Such research can be compared against our metagenomic and transcriptomic data during bloom as well as to previous metatranscriptomic sequencing (18) to better understand how or if the microbial community of Mono Lake returns to its previous state after extended periods of both monomixis and algal bloom.

## MATERIALS AND METHODS

### Sampling.

A vertical profile of photosynthetic active radiation (PAR) (2π quantum sensor; wavelength, 400 to 700 nm [Li-Cor]; energy [*E*] per square meter per second), dissolved oxygen (SBE 43 sensor; milligrams/liter), and attenuation coefficient (WetLabs Transmissometer; 600-nm wavelength light source, 10-cm path length; per meter) from surface (0 m) to ∼30 m was taken using a SeaBird SBE 19 conductivity, temperature, and depth (CTD) probe calibrated for use at Mono Lake. After measurements were obtained, water was pumped from depth to the surface at station 6 (lat 37.95739, long −119.0316) (Fig. 1) and sampled at 2 m, 10 m, 20 m, and 25 m the following day (due to unfavorable lake conditions) using a submersible well pump. Water was allowed to flow from the measured depth for 1 to 2 min to clear any residual water from the lines prior to sampling. Artemia organisms were removed from water samples using clean cheese cloth prior to filling 1-liter sterile high-density polyethylene (HDPE) containers. Samples were stored in a dark cooler until filtration occurred. Sediment was sampled at a 10-m depth approximately 6.9 kilometers away from the water transect (lat 37.9800, long −119.1048) using a box-core sampling device. Well water (lat 38.0922, long −118.9919) was sampled by allowing the wellhead to flow for approximately 5 min before a 5-liter HDPE container was completely filled. For influent stream water, 1 liter of water was taken from each location (Mill, lat 38.0230, long −119.1333; Rush, lat 37.8883, long −119.0936; Wilson, lat 38.0430, long −119.1191) into a sterile HDPE container. Lee Vining (lat 37.9422, long −119.1194) was sampled with the use of a submersible pump (as described above) into a sterile 1-liter HDPE container.

### Geochemical water analysis.

To characterize the water samples taken from 2 m to 25 m, major anions were measured using a Dionex ICS-90 ion chromatography system running an AS14A (4- by 250-mm) column. Major cations were also measured using a Perkin-Elmer Optima 5300 DV inductively coupled plasma optical emission spectrometer (ICP-OES). Both ion chromatography (IC) and ICP were conducted in the Department of Chemistry at the Colorado School of Mines. All sediment samples were extracted for IC and ICP analysis according to Florida Department of Environmental Protection method NU-044-3.12. All fluid samples were filtered in the field using 0.22-μm-pore-size polyethersulfone (PES) filters. All ICP samples were acidified with trace metal-grade nitric acid as per a standard procedure to ensure stabilization of all metal cations.

### Environmental sampling, field preservation, and DNA/RNA extraction of samples.

Immediately after sampling concluded, water from Mono Lake and surrounding streams was filtered onto 25-mm 0.22-μm-pore-size polyethersulfone filters (Merck Millipore Corp., Billerica, MA) in triplicate. Separate triplicate filters were obtained from each water sample for DNA and RNA extraction. Filter volumes are available in Table S1 in the supplemental material. After filtration, samples were immediately suspended in 750 μl of DNA/RNA shield (Zymo Research Co., Irvine, CA) and homogenized on site using a custom-designed lysis head for 1 min using a reciprocating saw. Sediment samples were immediately preserved on site by adding sediment directly to DNA/RNA shield as above. Preserved samples were maintained on dry ice and then stored at −80°C (RNA) or −20°C (DNA) until extractions were performed. DNA extraction was carried out using a Zymo Xpedition DNA minikit (Zymo Research Co.), and samples were eluted into a final volume of 100 μl. RNA extraction was performed using a Zymo Quick-RNA Miniprep kit (Zymo Research Co.) according to the manufacturer's instructions.

### rRNA gene sequencing library preparation.

Libraries of bacterial, archaeal, and eukaryotic small-subunit (SSU) rRNA gene fragments were amplified from each DNA extraction using PCR with primers (Integrated DNA Technologies Co., Coralville, IA) that spanned the rRNA gene V4 hypervariable region between position 515 and 926 (Escherichia coli numbering) that produced a ∼400-bp fragment for bacteria and archaea and a 600-bp fragment for the eukaryotes. These primers evenly represent a broad distribution of all three domains of life (17). The forward primer 515F-Y (**GTAAAACGACGGCCAG**CCGTGYCAGCMGCCGCGGTAA-3′) contains the M13 forward primer (in bold) fused to the SSU RNA gene-specific forward primer (underlined) while the reverse primer 926R (5′-CCGYCAATTYMTTTRAGTTT-3′) was unmodified from Parada et al. (17). 5 Prime Hot master mix (5 Prime, Inc., Gaithersburg, MD) was used for all reactions at a final volume of 50 μl. Reaction products were purified using AmpureXP paramagnetic beads (Beckman Coulter, Inc., Indianapolis, IN) at a final concentration of 0.8× (vol/vol). After purification, 4 μl of PCR product was used in a barcoding reaction, cleaned, concentrated, and pooled in equimolar amounts as previously described (29). The pooled, prepared library was then submitted for sequencing on an Illumina MiSeq platform (Illumina, Inc., San Diego, CA) using V2 PE250 chemistry.

### Quantitative PCR.

Total bacterial/archaeal and eukaryotic small-subunit (SSU) rRNA gene counts within the water column were obtained using two TaqMan-based probe assays as previously described (30, 31). Briefly, both assays were carried out using 25-μl reaction mixtures containing 1× the final concentration of Platinum Quantitative PCR SuperMix-UDG with 6-carboxy-X-rhodamine (ROX) (Thermo Fisher Scientific, Inc.), 1.8 μM each primer, and 225 nM bacterial/archaeal or eukaryotic probe. Further information related to the quantitative PCRs (qPCRs) carried out can be found in Table S5.

### SSU rRNA gene analysis.

Sequence reads were demultiplexed in QIIME, version 1.9.1 (32), and filtered at a minimum Q score of 20 prior to clustering. Sequence reads were first denoised and then clustered into operational taxonomic units (OTUs) at 97% similarity using UPARSE (33). After clustering, OTUs were assigned taxonomy using mothur (34) against the SILVA database (release 128 [r128]) (35). Each OTU was then aligned against the SILVA r128 database using pyNAST (36) and filtered to remove uninformative bases, and then a tree was created using the maximum likelihood method and the Jukes-Cantor evolutionary model within FastTree, version 2 (37). A BIOM-formatted file (38) was then produced for use in analyses downstream. To limit OTUs originating from contaminating microorganisms found in extraction and PCR reagents (39), all extraction blanks and PCR controls were processed separately, and a core microbiome was computed. Any OTU found in 95% of controls was filtered from the overall data set. Differences in community composition were estimated using the weighted UniFrac index (40). The effect of depth was tested with Adonis using the R package Vegan (41) within QIIME. Taxon heat maps and ordination plots were generated using phyloseq (42) and AmpVis (43).

A mapping file is available in Table S2. The mapping file, as well as BIOM files used for analyses, are available at https://zenodo.org/record/1247529, including an R Markdown notebook that lists the necessary steps to automate initial demultiplexing, quality filtering, and OTU clustering, as well as to reproduce figures associated with the rRNA gene analyses.

### Metagenomic/transcriptomic sequencing.

Metagenomic and metatranscriptomic samples were prepared using the Nextera XT library preparation protocol. Prior to library preparation, first-strand cDNA synthesis was carried out using a ProtoScript cDNA synthesis kit (New England BioLabs, Ipswich, MA), followed by second-strand synthesis using an NEBNext mRNA second-strand synthesis module (New England BioLabs). A mixture of random hexamer and poly(A) primers was used during first-strand synthesis. After conversion to cDNA, samples were quantified using a QuBit HS assay and then prepared for DNA sequencing. Briefly, 1 ng of DNA or cDNA was used as input into the NexteraXT protocol (Illumina, Inc.) according to the manufacturer's instructions. After amplification, libraries were cleaned using AmpureXP paramagnetic beads and normalized according to the NexteraXT protocol. All metagenomic and transcriptomic samples were then sequenced on an Illumina NextSeq 500 Instrument using PE150 chemistry (Illumina, Inc.) at the Oklahoma Medical Research Foundation (Oklahoma City, OK).

### Metagenomic assembly and binning.

Prior to assembly, metagenomic libraries were quality filtered, and adapters were removed using PEAT (44). A coassembly was produced using MEGAHIT (45) with a minimum contig length of 5,000 base pairs. After assembly, quality-filtered reads from triplicate individual samples concatenated to form single libraries per depth were mapped to the coassembly using Bowtie2 (42). Assembled contigs greater than 5 kb in length were first filtered to remove eukaryotic sequence using EukRep (46) and then binned into MAGs using CONCOCT (47) and refined using Anvi'o (48) in an attempt to manually reduce potential contamination or redundancy within each bin. Finally, bin quality was assessed using CheckM (49).

CheckM was also used to identify possible SSU rRNA gene fragments within each bacterial or archaeal bin. Putatively identified SSU rRNA gene fragments were aligned against the SILVA 132 database (35) using SINA (50). After alignment, sequences were added to the SILVA tree by SINA, and near relatives were included to give a putative identification of MAGs containing SSU sequence. Eukaryotic bins were checked for completeness with BUSCO (51).

### Metatranscriptomic analysis.

Metatranscriptome libraries were first filtered for quality and adapter removal using PEAT (44). After quality control, sequence files were concatenated into a single set of paired-end reads in FASTQ format and then assembled *de novo* using Trinity (52). Postassembly, the Trinotate package (https://trinotate.github.io/) was used to annotate assembled transcripts. After assembly, reads were quantified using Salmon (53), and differential significance was assessed using DEseq2 (54). Expression values were normalized as the number of transcripts per million, or TPM. Additionally, multiple housekeeping genes, including DNA gyrase (*gyrA* and *gyrB*), glyceraldehyde-3-phosphate dehydrogenase (GAPDH), and tubulin alpha were identified and used to determine if greater levels of overall transcription were identified at either sampled depth. Assembly, annotation, mapping, and statistical analyses were carried out using XSEDE compute resources (55).

### Accession number(s).

Sequence data are available in the NCBI Sequence Read Archive under the BioProject accession number PRJNA387610.

## Supplementary Material

Supplemental file 1

Supplemental file 2

Supplemental file 3

Supplemental file 4

Supplemental file 5
