# Supplementary material for: Metabolic Capability and Phylogenetic Diversity of Mono Lake during a Bloom of the Eukaryotic Phototroph Picocystis sp. Strain ML
Source: Appl Environ Microbiol. 2018 Oct 17;84(21):e01171-18. doi: 10.1128/AEM.01171-18 (PMC6193381; doi:10.1128/AEM.01171-18)
Supplement: Supplemental file 4 [file zam021188811s4.pdf]

Supplemental Figures S1 – S3, and Supplemental Table Legends

Supplemental Figure S1. Heatmap of the top 25 16S rRNA gene OTUs, including chloroplast, with percent relative abundance values shown for each labeled taxon. OTUs are named by Phyla, and the most likely genera.

Supplemental Figure S2. Phylogenetic tree of 16S rRNA gene sequences identified from MAGs within the bacterial and archaeal dataset. Accession numbers of near relatives are shown in addition to MAG numbers from which 16S sequence originated. The unbootstrapped tree was produced by adding aligned 16S rRNA gene sequence to the global SILVA tree within ARB, and removing unnecessary sequence to reduce tree size.

Supplemental Figure S3. KEGG metabolic map of genes identified within the putative *Picocystis* MAG (eukaryotic MAG 1). Identified enzymes and pathways are shown in green.

Supplemental Table S1. Water chemistry (ICP AES/IC) results from Mono Lake, well water, and Lee Vining, Mill, Rush, and Wilson creeks. Filter volumes (in mL) for all water samples.

Supplemental Table S2. 16S/18S rRNA gene sequencing mapping file and summary statistics of individual rRNA gene sequence libraries after quality control.

Supplemental Table S3. Summary statistics of metagenomic and transcriptomic assemblies. Identified genes within each assembly ("Gene hits") from 2, 20, 25 m and sediment samples. Summary statistics and putative identification of MAGs using CheckM, and identified genes within MAGs.

Supplemental Table S4. Identified transcripts across the *de novo* co-assembled metatranscriptome, including differential expression results. Avalible for download at http://dx.doi.org/10.6084/m9.figshare.6272159.

Supplemental Table S5. Minimum information for publication of quantitative real-time PCR experiments (MIQE) for 16S and 18S rRNA gene qPCR.

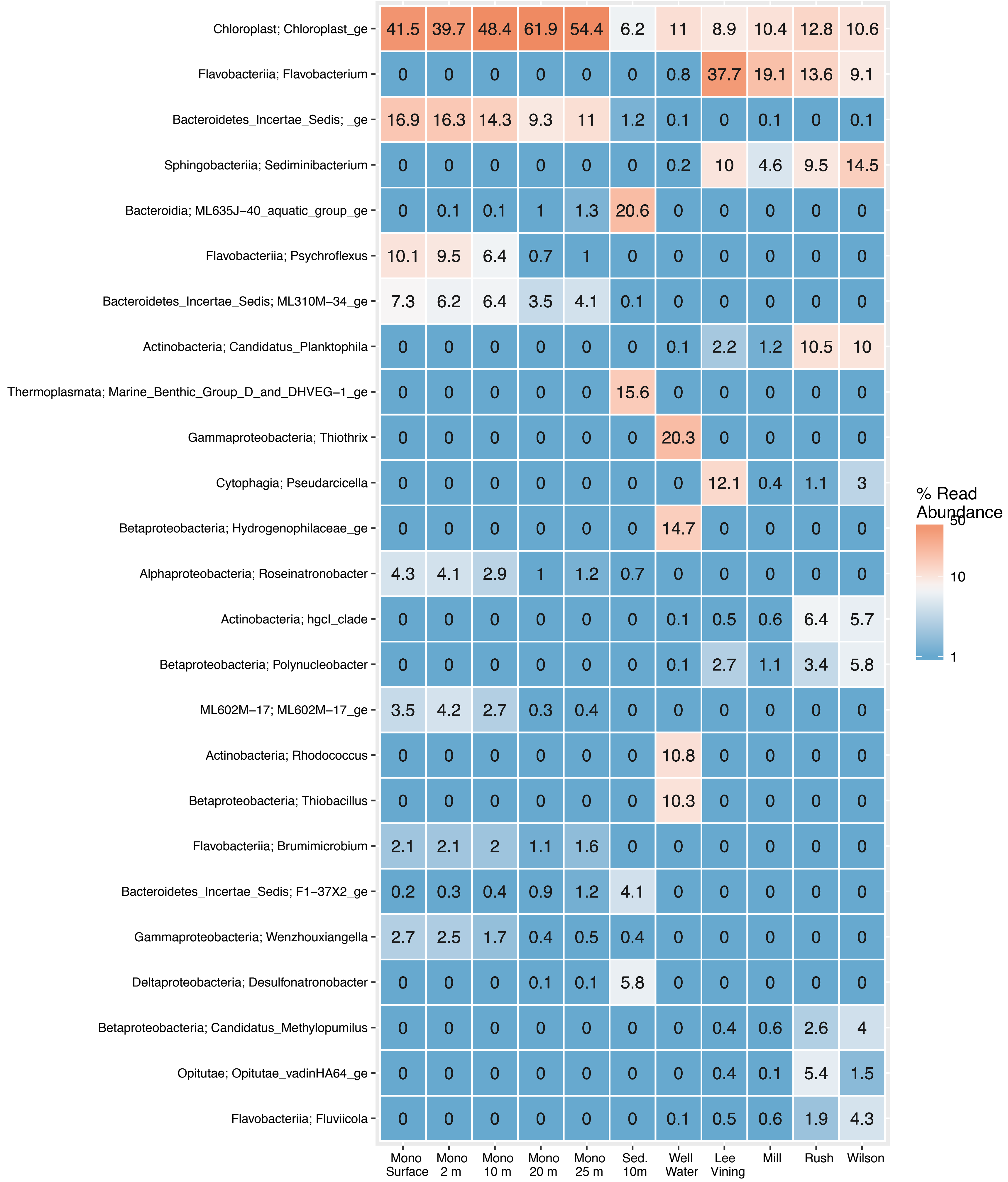
Chloroplast; Chloroplast_ge
41.5 39.7 48.4 61.9 54.4 6.2 11 8.9 10.4 12.8 10.6
Flavobacteriia; Flavobacterium
0 0 0 0 0 0 0.8 37.7 19.1 13.6 9.1
Bacteroidetes_Incertae_Sedis; _ge
16.9 16.3 14.3 9.3 11 1.2 0.1 0 0.1 0 0.1
Sphingobacteriia; Sediminibacterium
0 0 0 0 0 0 0.2 10 4.6 9.5 14.5
Bacteroidia; ML635J−40_aquatic_group_ge
0 0.1 0.1 1 1.3 20.6 0 0 0 0 0
Flavobacteriia; Psychroflexus
10.1 9.5 6.4 0.7 1 0 0 0 0 0 0
Bacteroidetes_Incertae_Sedis; ML310M−34_ge
7.3 6.2 6.4 3.5 4.1 0.1 0 0 0 0 0
Actinobacteria; Candidatus_Planktophila
0 0 0 0 0 0 0.1 2.2 1.2 10.5 10
Thermoplasmata; Marine_Benthic_Group_D_and_DHVEG−1_ge
0 0 0 0 0 15.6 0 0 0 0 0
Gammaproteobacteria; Thiothrix
0 0 0 0 0 0 20.3 0 0 0 0
Cytophagia; Pseudarcicella
0 0 0 0 0 0 0 12.1 0.4 1.1 3
Betaproteobacteria; Hydrogenophilaceae_ge
0 0 0 0 0 0 14.7 0 0 0 0
Alphaproteobacteria; Roseinatronobacter
4.3 4.1 2.9 1 1.2 0.7 0 0 0 0 0
Actinobacteria; hgcI_clade
0 0 0 0 0 0 0.1 0.5 0.6 6.4 5.7
Betaproteobacteria; Polynucleobacter
0 0 0 0 0 0 0.1 2.7 1.1 3.4 5.8
ML602M−17; ML602M−17_ge
3.5 4.2 2.7 0.3 0.4 0 0 0 0 0 0
Actinobacteria; Rhodococcus
0 0 0 0 0 0 10.8 0 0 0 0
Betaproteobacteria; Thiobacillus
0 0 0 0 0 0 10.3 0 0 0 0
Flavobacteriia; Brumimicrobium
2.1 2.1 2 1.1 1.6 0 0 0 0 0 0
Bacteroidetes_Incertae_Sedis; F1−37X2_ge
0.2 0.3 0.4 0.9 1.2 4.1 0 0 0 0 0
Gammaproteobacteria; Wenzhouxiangella
2.7 2.5 1.7 0.4 0.5 0.4 0 0 0 0 0
Deltaproteobacteria; Desulfonatronobacter
0 0 0 0.1 0.1 5.8 0 0 0 0 0
Betaproteobacteria; Candidatus_Methylopumilus
0 0 0 0 0 0 0 0.4 0.6 2.6 4
Opitutae; Opitutae_vadinHA64_ge
0 0 0 0 0 0 0 0.4 0.1 5.4 1.5
Flavobacteriia; Fluviicola
0 0 0 0 0 0 0.1 0.5 0.6 1.9 4.3
Mono Surface
Mono 2 m
Mono 10 m
Mono 20 m
Mono 25 m
Sed. 10m
Well Water
Lee Vining
Mill
Rush
Wilson
% Read Abundance
50
10
1

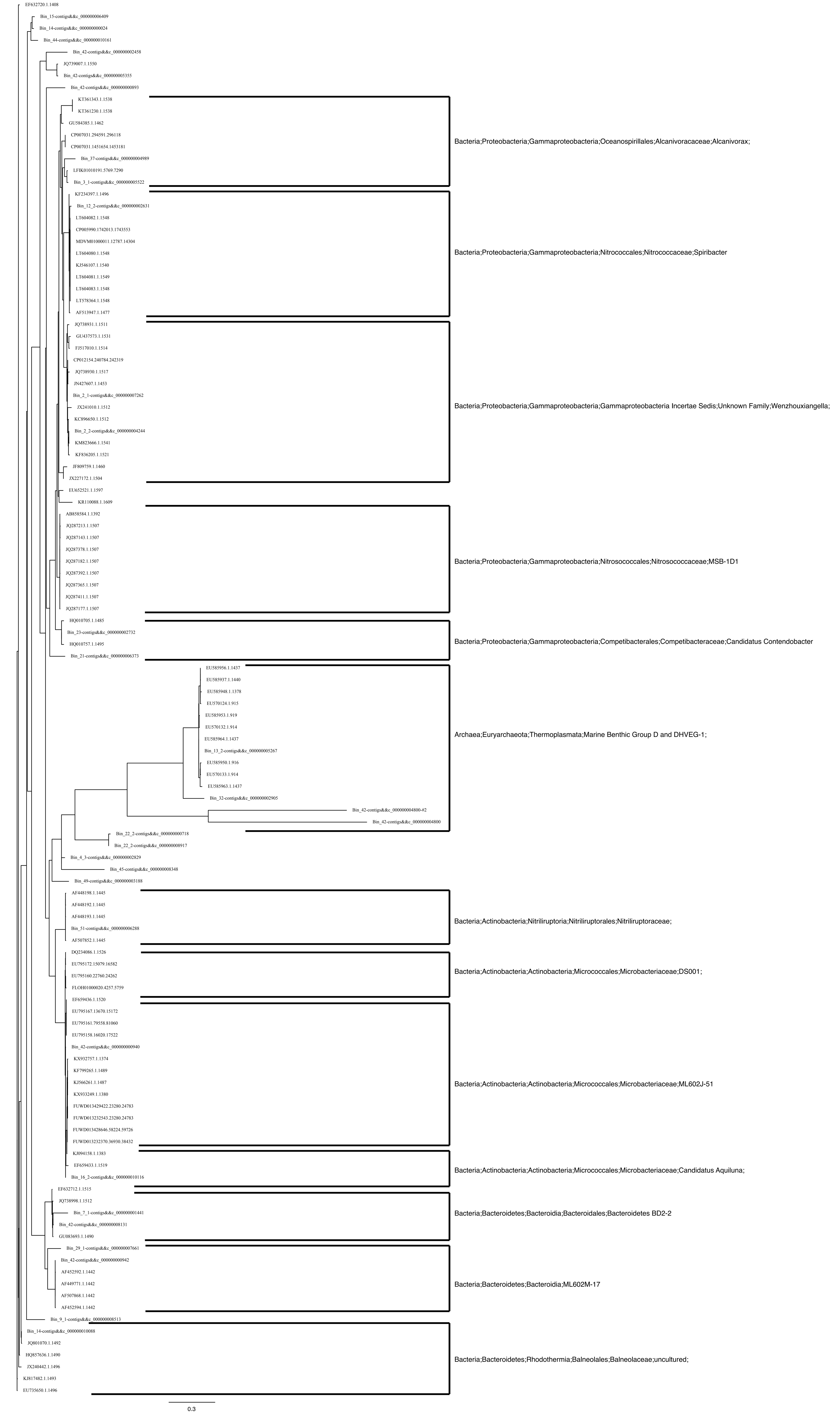

EF632720.1.1408
Bin_15-contigs&&c_000000006409
Bin_14-contigs&&c_000000000024
Bin_44-contigs&&c_000000010161
Bin_42-contigs&&c_000000002458
JQ739007.1.1550
Bin_42-contigs&&c_000000005355
Bin_42-contigs&&c_000000000893
KT361343.1.1538
KT361230.1.1538
GU584385.1.1462
CP007031.294591.296118
CP007031.1451654.1453181
Bin_37-contigs&&c_000000004989
LFIK01010191.5769.7290
Bin_3_1-contigs&&c_000000005522
Bacteria;Proteobacteria;Gammaproteobacteria;Oceanospirillales;Alcanivoracaceae;Alcanivorax;
KF234397.1.1496
Bin_12_2-contigs&&c_000000002631
LT604082.1.1548
CP005990.1742013.1743553
MDVM01000011.12787.14304
LT604080.1.1548
KJ546107.1.1540
LT604081.1.1549
LT604083.1.1548
LT578364.1.1548
AF513947.1.1477
Bacteria;Proteobacteria;Gammaproteobacteria;Nitrococcales;Nitrococcaceae;Spiribacter
JQ738931.1.1511
GU437573.1.1531
FJ517010.1.1514
CP012154.240784.242319
JQ738930.1.1517
JN427607.1.1453
Bin_2_1-contigs&&c_000000007262
JX241010.1.1512
KC896650.1.1512
Bin_2_2-contigs&&c_000000004244
KM823666.1.1541
KF836205.1.1521
JF809759.1.1460
JX227172.1.1504
Bacteria;Proteobacteria;Gammaproteobacteria;Gammaproteobacteria Incertae Sedis;Unknown Family;Wenzhouxiangella;
EU652521.1.1597
KR110088.1.1609
AB858584.1.1392
JQ287213.1.1507
JQ287143.1.1507
JQ287378.1.1507
JQ287182.1.1507
JQ287392.1.1507
JQ287365.1.1507
JQ287411.1.1507
JQ287177.1.1507
Bacteria;Proteobacteria;Gammaproteobacteria;Nitrosococcales;Nitrosococcaceae;MSB-1D1
HQ010705.1.1485
Bin_23-contigs&&c_000000002732
HQ010757.1.1495
Bin_21-contigs&&c_000000006373
Bacteria;Proteobacteria;Gammaproteobacteria;Competibacterales;Competibacteraceae;Candidatus Contendobacter
EU585956.1.1437
EU585937.1.1440
EU585948.1.1378
EU570124.1.915
EU585953.1.919
EU570132.1.914
EU585964.1.1437
Bin_13_2-contigs&&c_000000005267
EU585950.1.916
EU570133.1.914
EU585963.1.1437
Bin_32-contigs&&c_000000002905
Bin_42-contigs&&c_000000004800-#2
Bin_42-contigs&&c_000000004800
Archaea;Euryarchaeota;Thermoplasmata;Marine Benthic Group D and DHVEG-1;
Bin_22_2-contigs&&c_000000000718
Bin_22_2-contigs&&c_000000008917
Bin_4_3-contigs&&c_000000002829
Bin_45-contigs&&c_000000008348
Bin_49-contigs&&c_000000003188
AF448198.1.1445
AF448192.1.1445
AF448193.1.1445
Bin_51-contigs&&c_000000006288
AF507852.1.1445
Bacteria;Actinobacteria;Nitriliruptoria;Nitriliruptorales;Nitriliruptoraceae;
DQ234086.1.1526
EU795172.15079.16582
EU795160.22760.24262
FLOH01000020.4257.5759
Bacteria;Actinobacteria;Actinobacteria;Micrococcales;Microbacteriaceae;DS001;
EF659436.1.1520
EU795167.13670.15172
EU795161.79558.81060
EU795158.16020.17522
Bin_42-contigs&&c_000000000940
KX932757.1.1374
KF799265.1.1489
KJ566261.1.1487
KX933249.1.1380
FUWD013429422.23280.24783
FUWD013232543.23280.24783
FUWD013428646.58224.59726
FUWD013232370.36930.38432
Bacteria;Actinobacteria;Actinobacteria;Micrococcales;Microbacteriaceae;ML602J-51
KJ094158.1.1383
EF659433.1.1519
Bin_16_2-contigs&&c_000000010116
Bacteria;Actinobacteria;Actinobacteria;Micrococcales;Microbacteriaceae;Candidatus Aquiluna;
EF632712.1.1515
JQ738998.1.1512
Bin_7_1-contigs&&c_000000001441
Bin_42-contigs&&c_000000008131
GU083693.1.1490
Bacteria;Bacteroidetes;Bacteroidia;Bacteroidales;Bacteroidetes BD2-2
Bin_29_1-contigs&&c_000000007661
Bin_42-contigs&&c_000000000942
AF452592.1.1442
AF449771.1.1442
AF507868.1.1442
AF452594.1.1442
Bacteria;Bacteroidetes;Bacteroidia;ML602M-17
Bin_9_1-contigs&&c_000000008513
Bin_14-contigs&&c_000000010088
JQ801070.1.1492
HQ857636.1.1490
JX240442.1.1496
KJ817482.1.1493
EU735650.1.1496
Bacteria;Bacteroidetes;Rhodothermia;Balneolales;Balneolaceae;uncultured;
0.3

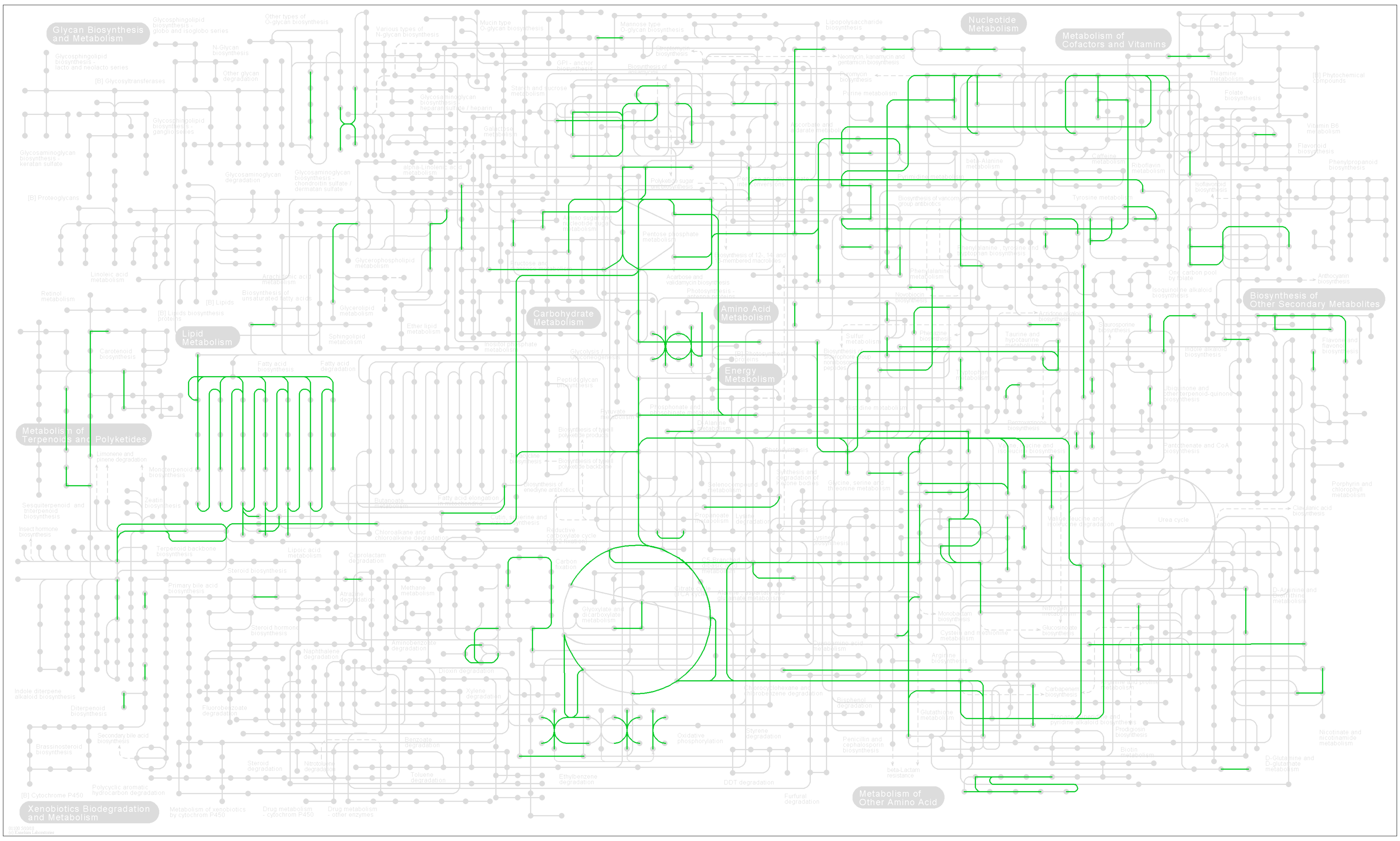
Glycan Biosynthesis and Metabolism
Nucleotide Metabolism
Metabolism of Cofactors and Vitamins
Biosynthesis of Other Secondary Metabolites
Carbohydrate Metabolism
Amino Acid Metabolism
Lipid Metabolism
Energy Metabolism
Metabolism of Terpenoids and Polyketides
Xenobiotics Biodegradation and Metabolism
Metabolism of Other Amino Acid
